# RNAi-mediated abrogation of trehalase expression does not affect trehalase activity in sugarcane

**DOI:** 10.1186/2193-1801-1-74

**Published:** 2012-12-21

**Authors:** Brian P O’Neill, Matthew P Purnell, Lars K Nielsen, Stevens M Brumbley

**Affiliations:** 1 Australian Institute for Bioengineering and Nanotechnology, The University of Queensland, Brisbane, Queensland 4072 Australia; 2 BSES Limited, PO Box 86, Indooroopilly, Queensland 4068 Australia; 3 Department of Biological Sciences, University of North Texas, 1155 Union Circle #305220, Denton, TX 76203-5017 USA

**Keywords:** RNA-interference, Saccharum, Sucrose-derivatives, Sugarcane biofactory, Trehalase, Trehalose

## Abstract

To engineer trehalose metabolism in sugarcane (*Saccharum* spp. hybrids) two transgenes were introduced to the genome: trehalose-6-phosphate synthase- phosphatase (TPSP), to increase trehalose biosynthesis and an RNAi transgene specific for trehalase, to abrogate trehalose catabolism. In RNAi-expressing lines trehalase expression was abrogated in many plants however no decrease in trehalase activity was observed. In TPSP lines trehalase activity was significantly higher. No events of co-integration of TPSP and RNAi transgenes were observed. We suggest trehalase activity is essential to mitigate embryonic lethal effects of trehalose metabolism and discuss the implications for engineering trehalose metabolism.

## Background

Trehalose is a two glucose disaccharide and is recognized by the US Food and Drug Administration as a safe food additive. Significant trehalose accumulation may not occur *in vivo* in angiosperms and other higher plants due to catabolism by trehalase (Glasziou and Gayler 1969). Trehalose metabolism – particularly its intermediate compound trehalose-6-phosphate (T6P) – plays a regulatory role in growth and carbon utilization (Paul et al. 2008; Smeekens et al. 2011) and is associated with abiotic stress tolerance in both native plants (viz. resurrection plants in arid landscapes) and metabolically engineered plants (Fernandez et al. 2010; Lopez-Gomez and Lluch 2012; Wingler 2002). In this study we investigated engineering trehalase activity to increase abiotic stress tolerance in sugarcane.

Numerous studies have increased abiotic stress tolerance in plants by over-expression of heterologous trehalose metabolic pathways; predominately trehalose-6-phosphate synthase (TPS, E.C.2.4.1.15) ± trehalose-6-phosphate phosphatase (TPP, E.C.3.1.3.12) from *E. coli*, yeast or *Arabidopsis*. Pleiotropic effects resulting in developmental abnormalities due to constitutive expression of these genes have been reported in tobacco, potato and tomato (Cortina and Culianez-Macia 2005; Jun et al. 2005; Yeo et al. 2000). T6P may be responsible for these effects because engineering strategies that minimize free T6P concentrations report the absence of negative pleiotropic effects.

Heterologous bacterial enzymes that synthesize trehalose independently of an intermediate have successfully increased abiotic stress tolerance in sugarcane and tobacco without negative pleiotropic effects on growth or development. Similar effects are observed due to tissue specific expression of heterologous TPS (± TPP) in rice and tobacco (Jang et al. 2003; Karim et al. 2007; Lee et al. 2003). Constitutive (i.e. ultimately cytosolic), chloroplast or stress inducible (ABA response) expression of TPSP (a fusion of *E. coli* TPS and TPP creating a single bi-functional enzyme, trehalose-6-phosphate synthase- phosphatase) in rice also caused no stunting of growth or other phenotypic abnormalities (Garg et al. 2002; Jang et al. 2003).

The regulatory effects mediated by the trehalose synthesis pathway are largely unknown. *Arabidopsis* TPS (*AtTps1*) null mutants indicate that T6P is required for *Arabidopsis* embryo development and glycolytic regulation in embryonic (Eastmond et al. 2002; Schluepmann et al. 2003) as well as vegetative tissues (Gomez et al. 2010). Schluepmann et al. (2012) propose a model of the growth regulation of T6P and carbon utilization via inhibition of the sucrose-nonfermentation1-related protein kinase1 (SnRK1) as demonstrated by Zhang et al. (Zhang et al. 2009). In sugarcane varieties engineered to over-produce isomaltulose, increased levels of T6P are associated with higher sucrose content via inhibition of SnRK1 (Wu and Birch 2010).

Trehalose metabolism affects starch biosynthesis. Exogenous trehalose increases ADP-glucose pyrophosphorylase (AGPase) activity and starch accumulation in the shoots of *Arabidopsis* (Fritzius et al. 2001; Wingler et al. 2000 and in isolated *Arabidopsis*plastids (Kolbe et al. 2005; Lunn et al. 2006). Furthermore, trehalose affects expression of the transcription factor ABI4 that is known to affects starch metabolism (Ramon et al. 2007). Trehalose metabolism may also act as a sugar sensor; T6P concentration is inversely related to sucrose concentration during carbon starvation and trehalose concentration has been shown to correlate with increasing sucrose concentration in sugarcane internodes during maturation (Glassop et al. 2007; Lunn et al. 2006). These data suggest that trehalose metabolism enables light-independent control of starch synthesis in response to sugar status. It may also provide a possible mechanism for how trehalose metabolism affects growth and carbon utilization whereas T6P acts as a signaling metabolite between the sucrose concentration in the cytosol and starch synthesis in the chloroplast.

In the present study, metabolic engineering of trehalose metabolism in sugarcane was investigated. To increase trehalose biosynthesis TPSP was over-expressed and attempts were made to abrogate trehalase activity using RNA-interference (RNAi). Results obtained suggest that trehalose biosynthesis and catabolism are co-ordinated to enable successful embryogenesis and that alternate trehalase activities are present in sugarcane. Overall, this work supports that trehalose metabolism acts as a sugar-sensing and signaling is supported.

## Results

Trehalose metabolism in sugarcane variety Q117 (*Saccharum* spp. hybrids) was engineered for value adding properties and to enhance abiotic stress tolerance. DNA constructs encoding the trehalose biosynthesis pathway (TPSP) and an RNA silencing vector targeting trehalase (the trehalose catabolic pathway) were introduced into the genomic DNA of embryogenic Q117 callus. Using these constructs, three engineering strategies were envisaged: TPSP and RNAi single transformants and TPSP + RNAi dual-transformants. To assess the metabolic effects of these transgenes, the integration, expression and activity of the enzymes was tested in the transgenic lines.

Plants were recovered that harbored either the TPSP or RNAi construct, but not both. The total transgenic population tested was TPSP transformants (*n* = 26) and RNAi transformants (*n* = 30). A further 45 *NptII* positive lines (the selection plasmid) were used as transgenic negative controls for comparison. The soluble carbohydrate content in both transgenic populations was not significantly different from the negative control population when comparing sucrose, glucose, fructose or trehalose (data not shown).

**Figure 1:**
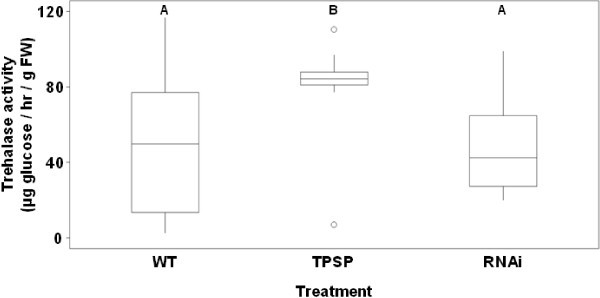
**Trehalase activity in young leaves.** Boxes represent the middle half of the data where the horizontal line is the median. Whiskers extend along the ‘typical’ range of data values. Probable outliers are represented as open circles. Statistically different populations are denoted with different capital letters (*P* < 0.05). The population sizes are: WT *n* = 30, TPSP *n* = 9 and RNAi *n* = 26. FW, fresh weight; Hr, hour; WT, wild type.

Trehalase activity was measured in young leaves of transgenic lines (Figure 1). Negative control type and RNAi positive lines (51.0 ± 7.3 and 46.9 ± 4.2 μg/hr/g FW, respectively) were not significantly different (*P* = 0.616) whilst TPSP positive lines (79.3 ± 9.6 μg/hr/g FW) had significantly increased trehalase activity compared to the negative control population (*P* = 0.047).

Trehalase activity was measured in an extended time course. This assay demonstrated that after 96 hours trehalase activity remained linear (91.5 ± 6 μg/hr/g FW). This result was verified using a porcine trehalase standard curve (0.002 U produced 152.5 μg/hr per assay). The trehalase inhibitor, validamycin A, was sufficient to wholly abrogate porcine trehalase activity however sugarcane trehalase activity was only reduced by 88.0 ± 1.0% (*n* = 6).

To ascertain the effects of the constructs on trehalase expression RT-PCR coupled with Southern blotting was used to identify the presence of the trehalase coding sequence (CDS) or the portion of the transcript encoding the pStarling 5^′^-untranslated region (5^′^-UTR) and the adjacent 5^′^ region of the trehalase CDS (Table 1). Total cDNA was synthesized from DNaseI treated RNA extracted from young leaves. Genomic DNA contamination was ruled out by performing RT-PCR with primers spanning an intron in β-actin that differentiated between genomic and mRNA amplification based upon a 331 bp size difference. The identity of the RT-PCR products were verified by Southern blots with a DIG-labeled nested primer specific for Treh-F. The trehalase CDS was identified in 77% of negative control lines (*n* = 22), 100% of TPSP lines (*n* = 22) and 37% of RNAi lines (*n* = 30). When Southern blots were performed on RT reaction products from the 5^′^-UTR – CDS primers 60% of RNAi lines were positive (*n* = 30).

**Table 1:** **Expression of the trehalase transcript and the pStarling-trehalase vector**

Transgene present in cDNA	#Lines	#Lines with RT product detected	RT product detected (%)
**Trehalase mRNA specific RT reaction**			
Negative Control	22	17	77
TPSP	22	22	100
pStarling-trehalase	30	11	37
**RNAi cassette 5′UTR/trehalase mRNA specific RT reaction**			
pStarling-trehalase	30	18	60

RT-PCR was performed using primers specific for the mRNA of trehalase and for a portion of the pStarling-trehalase construct containing the 5′ UTR and the trehalase coding sequence. RT products were blotted onto nitrocellulose membranes and screened with a gene specific probe. Expression was non-quantitatively assigned as positive or negative. RT, reverse transcription; UTR, untranslated region.

These experiments describe two strategies for the metabolic engineering of trehalose metabolism in sugarcane: 1) increasing the biosynthetic capacity through over-expression of heterologous enzymes and, 2) decreasing trehalose catabolic activity via abrogation of trehalase activity through RNAi. This paper describes the molecular characterization of these two transgenic populations. Novel sugarcane varieties produced via these approaches may have value-added properties and also superior abiotic stress tolerance characteristics. Our results provide evidence that trehalose metabolism is controlled by co-ordination of trehalose biosynthesis and its degradation via trehalase and that this system may have implications in regulation of embryogenesis.

## Discussion

Over-expression of trehalose biosynthesis in the absence of trehalase activity may be embryonic lethal. Single transformants with either TPSP or RNAi were viable however no plants were recovered with genomes that harbored both transgenes. It is known that *Arabidopsis* seedlings germinated on 100 mM trehalose have severely reduced growth (Paul and Pellny 2003) and that mature *Arabidopsis* plants accumulate 10 mM and 25 mM trehalose in a concentration dependent manner when cultured in the presence of trehalose (Wingler et al. 2000). Embryonic lethal AtTPS1 mutants are characterized by altered enzyme activities affecting carbon metabolism (Baud and Graham 2006) and the AtTPS1 gene affects expression of several genes involved in sugar signaling during plant development: disruption of its normal function in *Arabidopsis* causes multiple pleiotropic effects (Avonce et al. 2005). Therefore, it appears likely that trehalose metabolism affects embryonic development but otherwise does not perturb normal functions of mature plants.

The trehalase transcript was identified in all TPSP lines (but not all negative control plants) and these lines had significantly increased trehalase activities: Observations were made (data not shown) that trehalase activity in tobacco leaves increases when incubated in the presence of trehalose providing evidence that trehalose content affects trehalase activity. However because no changes in trehalose content were observed it is possible that these lines have increased trehalase activity to mitigate the effects of increased trehalose metabolism that otherwise would be embryonic lethal. The TPSP - RNAi phenotype appears to be embryonic lethal as we were unable to recover any dual-transformants; however a greater number of transformation attempts may be required to test this further.

Negative pleiotropic effects of engineered trehalose metabolism do not correlate with trehalose content. Therefore, whilst trehalose concentration may itself not be altered, flux through T6P, the trehalose intermediate compound, may be altered and the effects masked by trehalose catabolism. Therefore, increased trehalase activity in TPSP lines may be indicative of increased trehalose biosynthesis. T6P is known to affects embryogenesis and is a sugar signaling and regulatory molecule (Schluepmann et al. 2003; Schluepmann et al. 2004). Altered flux of this metabolite may lead to conditional lethality. Mutants of TPS1 display aberrant phenotypes thus a regulatory role of the protein itself may also be possible, as suggested in yeast (Eastmond et al. 2002; van Vaeck et al. 2001).

Abrogation of trehalase expression did not lead to a reduction in trehalase activity. Trehalase is a highly specific and stable enzyme (Alexander 1973). Residual translation of the trehalase transcript prior to a complete RNAi effect may enable a sufficient amount of enzyme to accumulate to produce the trehalase activity observed. In our study, trehalase activity was partially reduced in crude enzyme extracts of young leaves when incubated in the presence of validamycin A. Whilst the difference in inhibition may be attributed to competitive binding of the inhibitor to other targets within the crude enzyme extract, the concentration of inhibitor was effective to completely abrogating porcine trehalase activity with a higher activity than observed in sugarcane leaves thus this concentration should be sufficient to abrogate sugarcane trehalase activity. If contamination is ruled out because sodium azide was present in extracts, unknown trehalase activity not inhibited by validamycin A may be responsible for the production of glucose from trehalose as observed in the assay.

In assays on tobacco leaves, validamycin A is sufficient to reduce > 99% of trehalase activity (Goddijn et al. 1997). Comparatively, extracts prepared from flowers of *Arabidopsis* show a 10-fold reduction in trehalase activity in the presence of validamycin A whereas leaf and root extracts are completely inhibited (Muller et al. 2001). In *Lotus japonica* trehalase activity was reduced by 65% when plants were cultured in the presence of validamycin A (Lopez et al. 2006). These tissue specific trehalase activities suggest that trehalose metabolism is subjected to unknown variations in regulation and expression.

If trehalose biosynthesis is embryonic lethal then TPSP over-expressing lines may have been recovered that had elevated trehalose biosynthesis activity that was not high enough to cause lethality. Concurrently, RNAi lines that were recovered may have had reduced trehalase activity that was viable only in the presence of reduced trehalose biosynthesis activity. This suggests that TPS and trehalase activity are co-ordinately regulated.

The data presented suggest trehalose metabolism is regulated to enable successful embryogenesis and alternate trehalase activities are present in sugarcane, supporting the role of trehalose metabolism in sugar-sensing and signaling pathways. Our data suggest that the transgenes of interest have a molecular phenotype in engineered sugarcane lines although they do not correlate with soluble carbohydrate content or enzyme expression and activity of the genes of interest. Peer studies have demonstrated enhanced abiotic stress tolerance in sugarcane, tomato and rice that did not correlate with these properties (Cortina and Culianez-Macia 2005; Jang et al. 2003; Zhang et al. 2006). Because there is a correlation between sucrose and trehalose content and trehalose is known to effect carbon metabolism, then applications such as high-early sugar varieties may be able to exploit trehalose metabolism in situations where sucrose metabolism is engineered.

We conclude that the engineering of trehalose metabolism to impart value-adding properties requires strategies to circumvent the embryonic lethal phenotype of increased trehalose metabolism. Secondly, because trehalose biosynthesis and trehalase activity may be co-ordinately regulated and no negative pleiotropic effects were observed, future experiments will be conducted to determine if the RNAi transgene can enhance abiotic stress tolerance in sugarcane.

## Materials and methods

Sugarcane transformation was performed as per Chong et al. (2007). Embryogenic sugarcane callus was co-bombarded with constructs encoding TPSP, trehalase RNAi or TPSP + trehalase RNAi. pSB-TPSP and the RNAi vector, pStarling, were obtained under agreements from Myongji University (Republic of Korea) and the Commonwealth Scientific and Industrial Research Organisation (Canberra, Australia), respectively. The sugarcane CDS was compiled from expressed sequence tag (EST) clones via a BLAST_N search (http://www.ncbi.nlm.nih.gov/) using the putative rice trehalase gene as query (Genbank Accession Number NM_197396). EST CA142592 was obtained from the Institute of Chemistry, University of Sao Paulo (Brazil). A 314 bp region of the trehalase coding sequence was amplified via PCR using the following primers: forward arm, Treh-F, b53+ b60 (5^′^-GGATCCCAGCGGGTGCAGTCGGAG-3^′^ + 5^′^-GGCGCGCCCGCGCCAGTTGCTTCCAC-3^′^) and reverse arm, Treh-R, b55 + b56 5^′^-GGTACCCAGCGGGTGCAGTCGGAG-3^′^ + 5^′^-ACTAGTCGCGCCAGTTGCTTCCAC-3^′^). Each arm was sequentially sub-cloned to yield pStarling-trehalase. Genomic DNA transgene incorporation was confirmed by PCR. Custom oligonucleotide primers were supplied from Sigma-Genosys (Castle Hill, New South Wales, Australia)

Total RNA was extracted from young leaf lamina (~50 mg) using the RNeasy Plant Kit (Qiagen, Doncaster, Victoria, Australia), DNaseI treated with RQ1 RNase-Free DNase (Promega, Annandale, New South Wales, Australia) and cDNA synthesized using the Improm-II Reverse Transcription System (Promega). Amplification of gene specific products from cDNA used the PCR cycle: initial denaturation 95°C 2 mins, denature (95°C), anneal (55°C) and extend (72°C) for 15 seconds each for 72 cycles and final extension 72°C 10 mins). β–actin primers b100 + b101 (5^′^-GGGATGACATGGAGAAAATCTGGC-3^′^ + 5^′^-TGGATGGCTGGAAGAGGACC-3^′^) nested in the CDS spanning an intron were utilized as a positive control. The trehalase CDS was amplified using b53 + b60 and a vector-trehalase specific product amplified with b94 (5^′^-CGGAGCGCACACACACACAACCAGATCTCC-3^′^) + b60.

RT-PCR products were transferred from agarose gels to a Hybond XL membrane (GE Healthcare, Rydalmere, New South Wales, Australia) using a Biorad Model 785 Vacuum Blotter (Biorad, Gladesville, New South Wales, Australia) with 0.4 M NaOH transfer solution. A DIG system was used to detect DNA fragments of interest as per manufacturer’s instructions (Roche Applied Science, Castle Hill, New South Wales, Australia). The DNA probe - a 154 bp fragment specific to Treh-F (amplified with primers b98 + b99 5^′^-TCCTGTCCCGCTACTTCG-3^′^ + 5^′^-GCCAGTTGCTTCCACAGC-3^′^) - was synthesized using DIG-labeled dNTPs in an otherwise standard PCR of 100 μL final volume. The DIG-labeled probe was gel purified and quantified using a Qubit Fluorometer and DNA Quant-It BR Assay Kit (Invitrogen, Mount Waverly, Victoria, Australia).

The hybridization procedure was carried out as per manufactures instruction using a Hybaid oven (Thermo Scientific, Noble Park, Victoria, Australia). The CDP-star labeled membrane was affixed to the inside of a dark-room cassette and Kodak Biomax MS Film (Kodak, Collingwood, Victoria, New South Wales, Australia) placed on top. All steps involving film were conducted in a dark room under red-light conditions. The membrane and film were incubated in the dark for 1 minute. The film was exposed by washing in Kodak GBX Developer and Replenisher, and Kodak GBX Fixer and Replenisher then rinsed in water prior to air-drying.

Crude enzyme extracts were prepared from 1000 mg of leaf tissue as per Chong et al. (2007). Trehalase activity in crude enzyme extracts was measured as the production of glucose from the sole assay substrate, trehalose. Assays were performed in McIlvaine’s buffer (pH 6.2) and 5 mM trehalose at 30°C for 96 hours using dH_2_0 with sodium azide (0.02% w/v) to prevent contamination. The specificity and linear range of the reaction were tested via inhibition with 1 mM validamycin A (Scientifix, Cheltenham, Victoria, Australia) and in comparison to a porcine trehalase standard (Sigma-Aldrich, Castle Hill, New South Wales, Australia). Glucose production was analyzed using a Shimadzu HPLC system with a refractive index detector using a Shodex Sugar KS-01 S-DVB gel (300 mm × 7.8 mm) Carbohydrate column (Phenomenex, Lane Cove, New South Wales, Australia). Separation was performed via injecting 20 μL of sample and eluting with MilliQ water at 0.9 mL min^-1^ for 15 minutes at 65°C.

The Statistix 8.0 software package (Analytical Software, Tallahassee, Florida, USA) was used to analyze data. Significant differences were deemed to be present when *P* < 0.05. Data was analyzed using a one-way analysis of variance using the Tukey HSD method.

## Abbreviations

CDS: Coding sequence
EST: Expressed sequence tag
RNAi: RNA-interference
T6P: Trehalose-6-phosphate
TPP: Trehalose-6-phosphate phosphatase
TPS: Trehalose-6-phosphate synthase
TPSP: Trehalose-6-phosphate synthase- phosphatase
UTR: Untranslated region
